# The Effectiveness of closed kinetic chain exercises in individuals with knee osteoarthritis: A systematic review and meta-analysis

**DOI:** 10.1371/journal.pone.0322475

**Published:** 2025-05-02

**Authors:** Ammar Fadil, Qassim Ibrahim Muaidi, Mohamed Salaheldien Alayat, Nahla Ahmad AlMatrafi, Moayad Saleh Subahi, Mansour Abdullah Alshehri

**Affiliations:** 1 Department of Medical Rehabilitation Sciences, Faculty of Applied Medical Sciences, Umm Al-Qura University, Makkah, Saudi Arabia; 2 Department of Physical Therapy, College of Applied Medical Sciences, Imam Abdulrahman bin Faisal University, Dammam, Saudi Arabia; Prince Sattam bin Abdulaziz University, SAUDI ARABIA

## Abstract

**Introduction:**

The aims of this review was to investigate the effectiveness of closed kinetic chain exercise (CKCE) on pain, function, and proprioception in individuals with knee osteoarthritis (OA).

**Methods:**

Nine databases were searched up to December 2023. Randomized controlled trials (RCTs) examining the effects of CKCE in individuals with knee OA were included. The methodological quality was assessed using the PEDro scale, and the level of evidence was evaluated with the GRADE system. A random-effects meta-analysis was conducted to assess differences between treatment groups for the primary outcomes (pain and function). Effect sizes were calculated using standardized mean differences (SMDs) with 95% confidence intervals (CIs).

**Results:**

A total of 24 studies were included in the descriptive analysis, and 18 studies were included in the quantitative analysis (meta-analysis). The meta-analysis results indicated that CKCE treatment led to greater improvements in pain (SMD = −0.76; 95% CI: −1.51, −0.01) and function (SMD = −1.25; 95% CI: −1.88, −0.62) compared to no treatment. A subgroup meta-analysis showed that the combined treatment of CKCE and conventional physical therapy (CPT) resulted in greater improvements in pain (SMD = −1.18; 95% CI: −1.70, −0.67) and function (SMD = −1.27; 95% CI: −1.79, −0.75) compared to CPT alone. The risk of bias assessment revealed that two studies were of low quality, nine were of fair quality, and the remaining 13 were of high quality. The GRADE system indicated a low quality of evidence for the effects of CKCE on both pain and function.

**Conclusion:**

While CKCE shows promise in reducing pain and improving function in individuals with knee OA, the quality of evidence is considered low according to the GRADE system. Further high-quality RCTs with larger sample sizes are needed to confirm the effectiveness of CKCE in managing knee OA.

## 1. Introduction

Osteoarthritis (OA) is a disease that affects the entire joint structure, causing progressive damage to the articular cartilage in synovial joints, along with subchondral bone sclerosis, osteophyte formation, and mild persistent synovial inflammation [1]. In 1986, the American Rheumatism Association defined OA as “a group of overlapping disorders with different etiologies but similar biological, morphological, and clinical outcomes.” The prevalence of knee OA in the United States has recently increased due to rising body mass index and longer life expectancy, both of which increase joint loads, as well as wear and tear process [2]. In Saudi Arabia, the prevalence of OA is estimated at 53.3% in males and 60.9% in females, with ages ranging from 30 to 90 years and a mean age of 49 years [3].

The development of OA is primarily associated with two mechanisms: abnormal loading on normal cartilage or normal loading on abnormal cartilage. These mechanisms represent the main risk factors for knee OA [1]. The clinical features of knee OA include joint inflammation [4], articular cartilage degeneration [5], joint instability [6], pain, and functional loss [7]. These signs and symptoms limit activities of daily living (ADL) and can progress to significant functional impairment and disability [7]. Generally, short- and long-term goals for treating knee OA focus on reducing pain, minimizing inflammation and swelling, improving proprioception, and enhancing functional activity and quality of life [7]. There are several treatment options for knee OA include anti-inflammatory drugs, therapeutic exercises, weight management, and physical therapy modalities [8].

Evidence-based guidelines for knee OA management recommend various interventions, including closed kinetic chain exercises (CKCEs), open kinetic chain exercises (OKCEs), aerobic exercises, strength training, and weight management [9–15]. CKCEs are typically performed with the feet fixed to an object, generating compressive forces in the ankle, knee, and hip joints [16]. These exercises have been shown to enhance muscle strength, proprioception, and neuromuscular control in the lower extremities [16]. While CKCE offers potential biomechanical benefits, its clinical efficacy compared to other physical therapy modalities or no treatment remains unclear. Several systematic reviews have examined physical therapy’s role in OA management, but most have evaluated mixed exercise interventions without specifically focusing on CKCE. Moreover, methodological inconsistencies across studies have led to conflicting findings [17–21], with no standardized treatment protocol established. To address these gaps, a systematic review with meta-analysis is needed.

This study aimed to systematically review and conduct a meta-analysis on the effectiveness of CKCE in individuals with knee OA. Specifically, this review aimed to: (1) assess the effects of CKCE on pain reduction compared to no treatment or other physical therapy interventions; (2) assess its impact on functional improvement relative to no treatment or other physical therapy interventions; (3) determine whether CKCE enhances proprioception compared to no treatment or other physical therapy interventions; (4) examine the effects of combining CKCE with other physical therapy interventions to determine whether combined therapy provides greater clinical benefits than individual interventions alone; and (5) evaluate the methodological quality and overall level of evidence.

## 2. Methods

### 2.1. Study design

This study was a systematic review and meta-analysis. The systematic review protocol was registered in PROSPERO (registration number: CRD42022316670) and conducted in accordance with the Preferred Reporting Items for Systematic Reviews and Meta-Analyses (PRISMA) guidelines (S1 Table).

### 2.2. Study strategy

The searches were conducted across nine electronic databases from January 1, 2010, to December 31, 2023, to ensure the inclusion of the most recent and relevant evidence from the last decade, reflecting current advancements in knee OA management. The search strategy was based on the PICO framework. A systematic search was performed in the following databases: PubMed, MEDLINE, Web of Science, EBSCO, Wiley Online Library, Science Direct, Scopus, PEDro, and Cochrane (CENTRAL). The Medical Subject Headings (MeSH) terms used in the MEDLINE database included: weight-bearing exercise program, weight-bearing strengthening program, resistance training, and knee osteoarthritis. Additional terms used in the search were: closed kinetic chain exercise, CKCE, weight-bearing exercise, progressive loading exercise, knee osteoarthritis, knee osteoarthrosis, and knee joint degenerative disease. The search also included the Grey Literature Report, OpenGrey databases, and ResearchGate. Furthermore, references of all eligible articles were screened for relevant studies.

### 2.3. Eligibility criteria

Studies were eligible if they met the following criteria (S2 Table): randomized controlled trials (RCTs) or clinical trials that investigated CKCE in the treatment of individuals with knee osteoarthritis; CKCE applied to at least one group; participants of any age (18+), sex, or race; pain and function as primary outcomes or proprioception as a secondary outcome; and full text available in English. Studies were excluded if they were not clinical trials (e.g., observational, cross-sectional, or case-control studies); both groups received CKCE; there was no control group; outcome measures (e.g., pain, function, and proprioception) were not assessed; or the full text was unavailable (e.g., abstracts, conference papers).

### 2.4. Study selection

An initial screening of titles and abstracts was conducted to assess eligibility. Duplicates were removed using EndNote X9 software (Clarivate Analytics, Philadelphia, PA, USA). Full-text articles that met the inclusion criteria were reviewed. To minimize bias, two reviewers (AF and NAA) independently performed the screening and study selection. Any disagreements were resolved in consultation with a third reviewer (MSA).

### 2.5. Data extraction

Data extracted included participant characteristics (e.g., age and sex), sample size, study design, outcome measures (e.g., pain, function, and proprioception), interventions (type, duration, and frequency of exercises), follow-up assessments, and a summary of results. The mean and standard deviation (SD) of outcome measures (when available) for both experimental and control groups were extracted. For missing data, the primary authors of the relevant studies were contacted to request the data. Two reviewers (AF and NAA) independently collected the data, and any discrepancies were reviewed and resolved by a third reviewer (MSA).

### 2.6. Methodological quality

Two independent assessors (AF and NAA) evaluated the methodological quality of each study using the PEDro scale. This scale is considered an internally valid and reliable tool for assessing the risk of bias for RCTs and clinical trial, and is commonly used in systematic reviews [22,23]. The PEDro scale consists of 11 items (yes/no questions). The first item assesses external validity, while items 2–11 assess internal validity. Scores range from 0 to 10, and study quality is classified as low (≤3), fair (4–5), or high (>6). Any scoring discrepancies were resolved by consensus with a third reviewer (MSA).

### 2.7. Quality of evidence

The quality of evidence was evaluated using the Grading of Recommendations Assessment, Development, and Evaluation (GRADE) system [22], which provides a structured process for summarizing and presenting evidence. GRADE ratings range from very low to high quality and are based on five criteria: (1) study limitations, (2) inconsistency, (3) indirectness, (4) imprecision, and (5) publication bias [25]. Each criterion may be rated as having minimal, serious, or extremely serious limitations [25].

### 2.8. Data analysis

Qualitative (descriptive) data were presented in tables. Quantitative analysis was conducted through meta-analysis using RevMan software (version 5.3). Pain and function were considered primary outcomes, while proprioception was considered a secondary outcome in the meta-analyses assessing CKCE’s effectiveness in individuals with knee OA. A random-effects model was used, and overall and individual study effect sizes were estimated using standardized mean differences (SMDs) with 95% confidence intervals (CIs). Effect sizes were categorized as small (0.2), medium (0.5), or large (≥0.8) [23]. The percentage of total variability due to between-study heterogeneity was assessed using the I² index, which can be classified as low (I² ≤ 25%), moderate (I² 26–50%), or high (I² ≥ 75%) [24]. A subgroup meta-analysis was performed to compare the effects of CKCE to other treatments.

## 3. Results

### 3.1. Study selection

**Fig 1:**
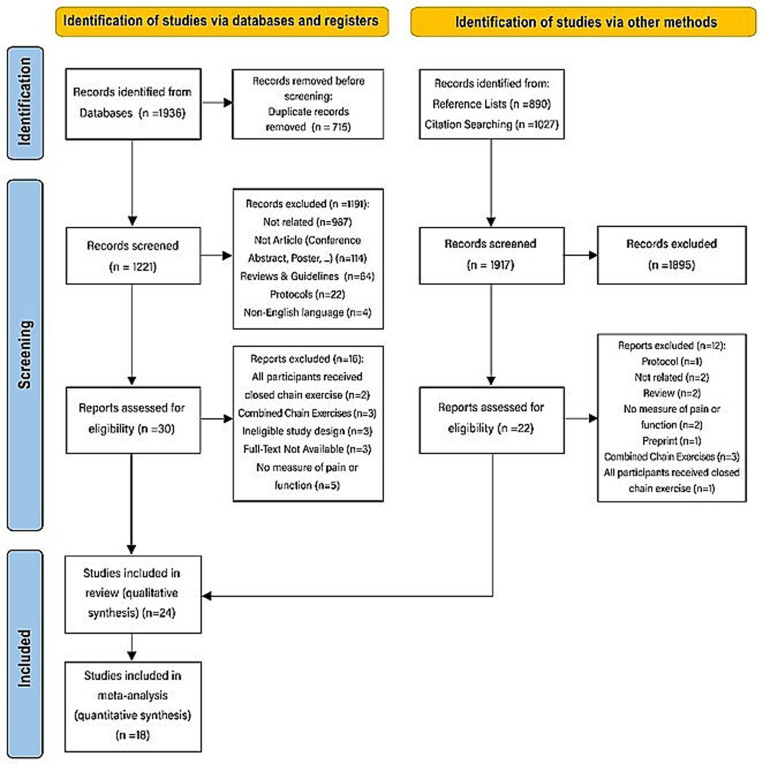
Flow diagram of the study selection process.

A total of 3,853 articles (1936 articles from databases and 1,917 articles from other sources; Fig 1 and S3-S4 Tables) were identified, of which 715 duplicates were removed. After reviewing the titles and abstracts of the remaining 3,138 articles, 3,086 irrelevant articles were excluded. Following full-text screening, 28 more articles were removed for not meeting the eligibility criteria, leaving 24 studies [17–21,25–43] included in this systematic review. Fig 1 illustrates the PRISMA flow diagram.

### 3.2. Study characteristics

This systematic review included 1,383 participants, with ages ranging from 30 to 80 years. The experimental group had 555 participants, while the control (no treatment or other treatment modalities) group had 627. Fourteen studies [17,20,21,25–27,30,34,35,37,38,40,42,43] included participants of both sexes (n = 894), with a male-to-female ratio of 232/662. Five studies [19,28,29,31,32] included only female participants (n = 223), while five studies [18,33,36,39,41] did not report the participants’ sex (n = 266). Sixteen studies [17,18,21,25–27,29,32,34–40,42] included individuals with mild-to-moderate knee OA, and one study [20] included individuals with a mixed presentation of knee OA (mild, moderate, and severe). Eight studies did not provide sufficient information regarding the grade of knee OA [19,28,30,31,33,41,43]. Table 1 shows the study characteristics.

**Table 1:** Study characteristics, outcome measures and results.

Study	Study design	Subjects (F/M) ratio	Measured variables	Intervention	Follow up	Results
Lin et al., 2007	RCT	81 participants assigned to: G1: n = 29; 20/9 G2: n = 26; 21/5 G3: n = 26, 21/5	1. knee proprioception 2.Physical function (WOMAC) 3. Walking speed	G1:(CPFE) G2: (CKCE) G3: Control (no exercise)	No	Both CPFE and CKCE programs were effective in improving all measured variables than control group.
Jan et al., 2008	RCT	98 participants assigned to: G1: n = 34; 27/7 G2: n = 34; 27/7 G3: n = 30; 25/5	1. Pain & physical function:(WOMAC) 2. Walking speed	G1: HR leg-press G2: LR leg-press G3: Control (no exercise)	No	Significant improvement for all measures was observed in both exercise groups, while no changes were found in the control group.
Jan et al., 2009	RCT	106 participants assigned to: G1: n = 36; 24/12 G2: n = 35; 25/10 G3: n = 35 24/11	1. Physical function (WOMAC) 2. Walking speed 4. knee proprioception	G1: WB G2: NWB G3: Control (no exercise)	No	Significant improvement for all measures was observed in both exercise groups, except for reposition error which was improved with non-significant difference in the NWB group.
Şahin et al., 2010	RCT	45 female participants assigned to: G1: n = 18 G2: n = 17 G3: n = 10	1. WOMAC 2. Chair-stand test 3. 6MWT 4. Stairs descent and stairs climbing 5. Static and dynamic balance	G1: OKCE G2: CKCE G3: Control (no exercise)	No	Significant improvement for All measures in both exercise groups, there was no significant difference between OKCE and CKCE groups.
Chang et al., 2012	RCT	41 female participants assigned to: G1: n = 24 G2: n = 17	1. WOMAC 2. FFRT 3. 10-MWT 4. CS-30 5. TUG 6. GUD-13	G1: Elastic-bands leg-press exercise + physical therapy modalities G2: Physical therapy modalities	No	Statistically significant improvements in all measures were observed in the leg-press exercise group, except for the FFRT and GUD-13.
Simão et al., 2012	RCT	31 participants assigned to: G1: n = 10; 8/2 G2: n = 10; 9/ G3: n = 11; 10/	1. WOMAC 2. 6-MWT 3. BBS 4. Gait speed test 5. inflammatory markers	G1: SE-WBV G2: SE G3: Control (no training)	No	In the SE-WBV group, there was significant improvement compared with the control group.
Verma, 2012	Comparative study	30 female participants assigned to: G1: n = 15 G2: n = 15	1. WOMAC 2. Muscle strength	G1: CKCE + hot pack G2: OKCE + hot pack	No	CKCE group displayed significantly greater improvements compared with the OKCE.
Daskapan et al., 2013	RCT	40 female participants assigned to: G1: n = 20 G2: n = 20	1. VAS 2. TUG 3. Isokinetic muscle strengths 4. Static standing balance 5. KOOS-PS	G1: SLRE + electrotherapy G2: MSE + electrotherapy	1 month	The MSE group had significantly higher TUG score, and right knee extensor torque compared to the SLRE group. However, no significant between-groups difference was found in VAS, static balance, KOOS-PS.
Sudhesh, 2013	RCT	30 participants assigned to: G1: n = 15 G2: n = 14 F/M ratio not reported.	1. WOMAC 2. Q-angle	G1: CKCE + taping G2: Traditional approach	No	CKCE + taping group showed significant improvement when compared with the traditional approach group.
Shah, 2014	Clinical Trial	30 participants assigned to: G1: n = 15 G2: n = 14 F/M ratio not reported.	1. VAS 2. WOMAC	G1: OKCE + CPT G2: CKCE + CPT	No	There was no significant difference between CKCE and OKCE in reducing pain and improving Function.
Dinçer et al., 2016	RCT	30 participants assigned to: G1: n = 16; 13/3 G2: n = 14; 11/3	1. VAS 2. Function: (WOMAC) 3. Cartilage volume and thickness	G1: CKCE + CPT G2: CPT	No	Both groups improved significantly in pain and function, while the cartilage volume and thickness didn’t change significantly.
Olagbegi et al., 2016	RCT	83 participants; 51/32) assigned to: G1: n = 28 G2: n = 27 G2: n = 28	1. VAS 2. IKHOAM	G1: OKCE G2: CKCE G3: CCE	No	CCE is more effective for pain relief, however, there was non-significant between-group difference in functional improvement.
Nahayatbin et al., 2018	Comparative Study	48 participants assigned to: G1: n = 16 G2: n = 16 G3: n = 16 F/M ratio not reported	1. KOOS 2. 6-MWT	G1: CKCE + CPT G2: TCE + CPT G3: CPT	1 month	There was no significant difference between TCE and CKCE groups in the 6MWT. KOOS was significantly greater in the TCE group compared with CKCE and CPT
Adegoke et al., 2019	Quasi-experimental study	29 participants; 24/5 assigned to: G1: n = 15 G2: n = 14	1. VAS 2. FIQ 3. AROM + PROM	G1: OKCE G2: CKCE	No	There was a significant in both groups with non-significant difference between groups.
Baireddy et al., 2019	Comparative Study	60 female participants divided into three groups with 20 participants in each group.	1. VAS	G1: OKCE G2: CKCE G3: CCE	No	Statistically significant improvement in all groups was found, but the CCE group improved more than OKCE & CKCE groups.
Bennell et al., 2020	RCT	128 participants assigned to: G1: n = 62; 45/17 G2: n = 66; 41/25	1. NRS 2. WOMAC	G1: WB G2: NWB	No	There was no evidence of a between-group difference in pain or function, with both groups reporting improvements
GİRGİN et al., 2020	RCT	38 participants assigned to: G1: n = 19; 15/4 G2: n = 19; 17/2	1. VAS 2. WOMAC 3. SF-36	G1: OKCE + CPT G2: CKCE + CPT	No	There was no statistically significant difference between the groups.
Hejdysz et al., 2020	Pilot study	66 participants (49/17) assigned to**:** G1: n = 22 G2: n = 22 G3: n = 22	1. VAS 2. WOMAC 3. ROM	G1: CKCE G2: MT G3: Control (No therapy)	No	Both MT and CKCE groups showed significant improvement in WOMAC and VAS.
Lai et al., 2021	RCT	81 participants assigned to: G1: n = 27; 25/2 G2: n = 27; 22/5 G3: n = 27; 23/4	1. VAS 3. Knee proprioception test 4. TUG 5. 6-MWT	G1: ST G2: ST-WBV G3: Health education (HE)	No	No significant difference among groups was found in all measures except the isokinetic strength of knee extensors was improved significantly in ST-WBV compared with ST or HE.
Meenakshi et al., 2021	Comparative study	68 participants divided into two groups with 34 participants in each group (F/M ratio not reported)	1. VAS 2. Knee flexors and extensors strength 3. WOMAC	G1: Pilates exercises G2: CKCE	No	Both groups showed significant improvement in all measures.
Moreira et al., 2021	Randomized trial	30 participants assigned to: G1: n = 14; 9/5 G2: n = 16; 9/7	1. PPT 2. 1RM test 3. WOMAC	G1: WB G2: NWB	No	Both groups showed significant improvements, with non-significant between-groups differences.
Krupa and Dinesh, 2021	Comparative study	90 participants divided into 3 groups; (F/M ratio not reported	1. NRS 2. WOMAC 3. ROM	G1: OKCE + Hot pack G2: CKCE + Hot pack G3: CCE + Hot pack	No	Result showed that CCE is more effective than OKCE and CKCE alone.
Bhatnagar et al., 2022	Comparative study	40 participants assigned to: G1: n = 20; 12/8 G2: n = 20; 14/6	1. VAS 2. MMT 3. KOOS-PS	G1: MS + CPT G2: SLRE + CPT	No	Significant improvement for all measures was observed in both exercise groups. MS group was significantly improved more than the SLR group.
Desai et al., 2022	Quasi-experimental Study	30 participants assigned to: G1: n = 15; 5/10 G2: n = 15; 8/7	1. WOMAC 2. Y-balance test	G1: CKCE G2: OKCE	No	There was a significant improvement in dynamic balance and WOMAC in both groups. However, between groups analysis showed that CKCE displayed significantly greater effect.

RCT: Randomized control trial, F: Female, M: Male, G: Group, WOMAC: the Western Ontario and McMaster Universities Arthritis Index, CPFE: Computerized proprioception facilitation exercise, HR: High resistance, LR: Low resistance, 6MWT: Six-minute walk test, FFRT: Functional forward-reach test, 10MWT: 10-m walk test, CS-30: 30-s chair stand test, TUG: Timed Up and Go Test, GUD-13: Going up-and-down 13-stair test, BBS: Berg Balance Scale, SE: Squat exercise, SE-WBV: Squat exercise with whole-body vibration, VAS: Visual analogue scale, KOOS: Knee Injury and Osteoarthritis Outcome Score, KOOS-PS: Knee Injury and Osteoarthritis Outcome Score-Physical Function Short Form, SLRE: Straight led raising exercise, MS: Mini-squat, CPT: Conventional physical therapy, IKHOAM: Ibadan Knee/Hip Osteoarthritis Outcome Measure, TCE: Tai Chi exercises, FIQ: Functional index questionnaire, AROM: Active Range of Motion, PROM: Passive Range of Motion, NRS: Numerical rating scale, SF-36: Short Form Health Survey, PPTs: Pressure pain thresholds, ST: Static Squat, ST-WBV: Static squat training with whole body vibration, 1RM test: One-repetition maximum strength test, MT: Manual Therapy, MMT: Manual muscle test, CKCE: Closed kinetic chain exercise, OKCE: Open kinetic chain exercises, CCE: Combined chain exercises, WB: Weight-bearing exercise, NWB: Non-weight-bearing exercise.

### 3.3. Outcome measures

Pain was evaluated in 20 studies [17–21,27–30,32,34–43] using various tools, including the Visual Analog Scale (VAS) [17–19,21,32,34,35,37–39,42] (n = 11), Numeric Pain Rating Scale (NRS) [20,41] (n = 2), Western Ontario McMaster Universities Arthritis Index (WOMAC) subscale [27–30,43] (n = 5), Knee Injury and Osteoarthritis Outcome Score (KOOS) subscale [36] (n = 1), and pain pressure threshold (PPT) [40] (n = 1). Four studies did not assess pain [25,26,31,33]. Function was evaluated in 22 studies, using tools such as the WOMAC subscale [18,20,25–31,33,34,37–41,43] (n = 17), KOOS subscale [32,42] (n = 2), Ibadan Knee/Hip Osteoarthritis Outcome Measure (IKHOAM) [35] (n = 1), Functional Index Questionnaire (FIQ) [17] (n = 1), and the Six-Minute Walk Test (6-MWT) [21,36] (n = 2). One study did not assess function [19]. Proprioception was assessed in only three studies [21,25,26] via joint repositioning error tests. Table 1 demonstrates the outcome measures used for each study and main results.

### 3.4. Interventions

In the experimental group, 14 studies [17,19–21,25–28,30,35,38–40,43] used CKCE as the sole intervention, while 10 studies [18,29,31–34,36,37,41,42] combined CKCE with conventional physical therapy (CPT). CPT included a variety of therapeutic modalities, such as hot packs, stretching exercises, and electrotherapy. In the control group, six studies [19,20,28,39,40,43] used OKCE, nine studies [17,18,31–33,35,37,41,42] used CPT, and 10 studies [21,25–30,34,36,38] had no treatment group. The intervention durations varied from 10 days to 12 weeks. Only two studies provided follow-up data after one month of intervention [32,36], while the others did not report follow-up data. Table 2 shows the prescription of each intervention used in the included studies.

**Table 2:** Exercise prescription in the included studies.

Author	Exercise	Intensity	Weeks	No. of sessions	Session time
Lin et al., 2007	Shuttle Mini Clinic resistance device for CKCE.	10% of (BW). 5% added every 2 weeks. 10 sets of 10 repetitions and 1 min rest between sets.	8	3 sessions/week	Not reported
Jan et al., 2008	A leg-press machine was used to perform the CKCE.	HR group: 60% of 1RM, 3 sets of 8 repetitions LR group: 10% of 1RM, 10 sets of 15 repetitions Every 2 weeks, weight increased by 5%.	8	3 sessions/week	HR: 30min LR: 50min
Jan et al., 2009	EN-Dynamic resistance device was used to perform the CKCE.	50% of 1RM, 4 sets of 6 repetitions. and increased by 5% every 2 weeks.	8	3 sessions/week	Not reported
Şahin et al., 2010	Hip adduction and abduction, external rotation and internal rotation, knee flexion and extension and squat.	Two sets of 10 repetitions for each leg then increased to 14 repetitions per set. First two weeks, exercises were performed without an elastic band, then loads of 50% of 20 RM were used during the second 2 weeks and increased progressively.	12	2 sessions/week	Not reported
Chang et al., 2012	Elastic-bands leg-press exercise:	10 repetitions/set × 3 sets/ session	8	2 to 3 sessions/week	20 min
Simão et al., 2012	Squat exercise at approximately 10° to 60° of knee flexion.	The intensity of squat training was increased by increasing the time and number of sets	12	3 sessions/week	Not reported
Verma, 2012	Seated leg-press, rowing machine exercise, step up and down exercise, progressive jumping exercise on mini trampoline, and stationary biking	Not reported	5	3 sessions/week	Not reported
Daskapan et al., 2013	Mini squat:15°–20° knee flexion and hold for three-four seconds,	Participants performed 20 mini squats twice daily, then increased by five every two days	3	5 sessions/week	Not reported
Sudhesh, 2013	Mini squat: 0°–60° knee flexion.	8–10 repetitions/set × 2 sets/session.	3	Not reported	Not reported
Shah, 2014	Static quadriceps exercise in standing, Partial squatting exercise, Step-up & down and lateral Step up & down exercise, Foreword and sideways lunges, Single leg standing	10 repetitions/set × 3 sets/session	8	6 sessions/week	Not reported
Dinçer et al., 2016	Squat exercise: 0–60° knee flexion and hold for 3 seconds.	15 repetitions	12	5 sessions/week for 3 weeks,	30 min
Olagbegi et al., 2016	Quadriceps Setting Exercise, Wall slides, Step-up and step-down	Each exercise performed 10 repetitions.	12	3 sessions/week	Not reported
Nahayatbin et al., 2018	Standing terminal extension, mini squat (15° flexion), front and side step-up, and lunge exercise	All exercises were performed with a ten-sec hold and ten-sec rest, except the front and side step-up.	4	3 sessions/week	20 min
Adegoke et al., 2019	Quadriceps setting exercise, Mini-squats, and Step-up and step-down	Each exercise was done 10 repetitions/ session.	8	Not reported	Not reported
Baireddy et al., 2019	Seated leg presses, stationary bicycling, and step up – step down.	3 seconds rest was given between each repetition	6	Not reported	Not reported
Bennell et al., 2020	Sliding, stepping, wall push, crab walking, wall squat	3 sets of 10 repetitions, except for hip muscle strengthening exercises were varied.	12	4 times/ week for 12 weeks	Not reported
GİRGİN et al., 2020	Mini squat 30° knee flexion, Standing on one-foot, Active stretching exercise for hamstring muscle, Single toe rise, Wall push.	Each movement held for 10 seconds and 10 repetitions. The stretching movement was held for 15 seconds and 5 repetitions	6	5 sessions/week for 3 weeks	30 min
Hejdysz et al., 2020	Balance disc exercise and rolling the ball against the wall	Not reported	~ 1.5	1 session/day	Not reported
Lai et al., 2021	Participants performed static squat training with bent knees 30°–60°.	Duration time, sets and total time were increased progressively over the 8-week training period	8	3 sessions/week	12–39 min
Meenakshi et al., 2021	Week 1: Standing on one leg with leaning in all directions. Week 2: week 1 exercises + exercise with wobble board, sitting down and standing up from a low chair, plyometric exercise, etc. Week 3: week 2 exercises + walk heel-to-toe on soft ground	In the first three weeks, progression was done by adding new advanced exercises each week, then through the remaining weeks, the exercises from the third week were repeated.	6	3 sessions/week	Not reported
Moreira et al., 2021	A leg press machine was used to perform the CKCE. During the training period, the ROM was 0–40° flexion.	50% of 1RM and it increased by 5% every 2 weeks. Each session consisted of 4 sets/ 6 repetitions.	8	3 sessions/week	50 min
Krupa and Dinesh, 2021	Rowing machine exercise, standing wall slides, lunges, and step up-step down exercise.	Each exercise was done in 3 sets of 10 repetition.	2	1 session/day	Not reported
Bhatnagar et al., 2022	Single leg mini squat15–20° and hold for 3–4 seconds.	20 repetitions twice a day and every two days five repetitions increases were done as a progression.	3	5 sessions/week	Not reported
Desai et al., 2022	Mini-squats, step-ups, and step-down (forward and backward), lunges, and standing wall slides	Exercise progression was done weekly either by increasing repetitions or adding weights.	4	Not reported	Not reported

BW: Body weight, HR: High-resistance, LR: Low-resistance, RM: Repetition Maximum.

### 3.5. Risk of bias assessment

The methodological quality of the included studies, assessed using the PEDro scale, ranged from 3/10–8/10. Nine studies were rated as high quality [20,21,26–28,30,34,40,43], 13 studies as fair quality [17,25,29,31–33,35–39,41,42], and two studies as low quality [18, 19]. Detailed results are presented in Table 3.

**Table 3:** Methodological quality assessment of included studies (PEDro scale).

Study	Items											Total score for each study (10)
**Eligibility criteria ** ^*^	**Random allocation**	**Concealed allocation**	**Baseline comparability**	**Blind subjects**	**Blind therapists**	**Blind assessors**	**Adequate follow-up**	**Intention-to-treat analysis**	**Between-group comparisons**	**Point estimates and variability**	
Lin et al., 2007	Y	Y	N	Y	N	N	N	Y	N	Y	N	4
Jan et al., 2008	Y	Y	N	Y	N	N	Y	Y	Y	Y	Y	7
Jan et al., 2009	N	Y	N	Y	N	N	Y	Y	Y	Y	Y	7
Şahin et al., 2010	Y	Y	Y	Y	N	N	Y	Y	N	Y	Y	7
Chang et al., 2012	Y	Y	N	Y	N	N	N	N	N	Y	Y	4
Simão et al., 2012	N	Y	Y	Y	N	N	Y	Y	N	Y	Y	7
Verma, 2012	Y	Y	N	Y	N	N	N	Y	N	Y	Y	5
Daskapan et al., 2013	Y	Y	Y	N	N	N	N	Y	N	Y	Y	5
Sudhesh, 2013	N	Y	N	Y	N	N	N	Y	N	Y	Y	5
Shah, 2014	Y	Y	N	N	N	N	N	Y	N	Y	N	3
Dinçer et al., 2016	Y	Y	Y	Y	N	N	Y	Y	N	Y	Y	7
Olagbegi et al., 2016	Y	Y	N	Y	N	N	N	Y	N	Y	Y	5
Nahayatbin et al., 2018	N	Y	N	Y	N	N	N	Y	N	Y	Y	5
Adegoke et al., 2019	Y	N	N	Y	N	N	N	Y	N	Y	Y	4
Baireddy et al., 2019	Y	N	N	N	N	N	N	Y	N	Y	Y	3
Bennell et al., 2020	Y	Y	Y	Y	N	N	Y	Y	Y	Y	Y	8
GİRGİN et al., 2020	Y	Y	N	Y	N	N	N	Y	N	Y	Y	5
Hejdysz et al., 2020	Y	Y	N	Y	N	N	N	Y	N	Y	Y	5
Lai et al., 2021	Y	Y	Y	Y	N	N	Y	N	Y	Y	Y	7
Meenakshi et al., 2021	Y	Y	N	N	N	N	N	Y	N	Y	Y	4
Moreira et al., 2021	Y	Y	Y	Y	N	N	Y	N	Y	Y	Y	7
Krupa and Dinesh, 2021	Y	Y	N	N	N	N	N	Y	N	Y	Y	4
Bhatnagar et al., 2022	Y	Y	Y	N	N	N	N	Y	N	Y	Y	5
Desai et al., 2022	Y	Y	N	Y	N	N	N	Y	Y	Y	Y	6

*Not included in the final score.

### 3.6. Quality of evidence

To evaluate the level of evidence (Table 4), studies were categorized by intervention type. The comparison between experimental and control treatments was subdivided into CKCE vs. no treatment and CKCE vs. other treatments. GRADE indicated a low level of evidence for improvements in pain and function, primarily due to study limitations (e.g., low quality on the PEDro scale), inconsistencies (e.g., high heterogeneity), imprecision (e.g., wide confidence intervals with small sample sizes), and publication bias (e.g., inclusion of only positive results).

**Table 4:** Quality of evidence (GRADE).

Intervention	Outcome measured	N. of part. (studies)	Study limitation	Inconsistency	Indirectness	Imprecision	Publication bias	Overall quality of evidence	Effect Estimate SMD [95% CI]	Effect size	Direction
**CKCE vs No treatment**	**Pain**	144 (3)	Not Serious	Serious	Not Serious	Serious ^c^	Not Serious	Low ⨁⨁ⲞⲞ	−0.76 [−1.51, −0.01]	Medium	CKCE
	**Function** **(The less score the better results)**	144 (3)	Not Serious	Serious	Not Serious	Serious ^c^	Not Serious	Low ⨁⨁ⲞⲞ	−1.25 [−1.88, −0.62]	Large	CKCE
**CKCE vs Corresponding control**	**Pain**	570 (12)	Serious ^a^	Serious	Not Serious	Not Serious	Not Serious	Low ⨁⨁ⲞⲞ	−0.18 [−0.55, 0.19]	Small	CKCE
	**Function** **(The less score, the better results)**	537 (11)	Serious ^a^	Serious	Not Serious	Not Serious ^c^	Not Serious	Low ⨁⨁ⲞⲞ	−0.74 [−1.23, −0.26]	Medium	CKCE
	**Function** **(The more score, the better results)**	156 (4)	Serious ^a^	Serious	Not Serious	Serious ^c^	Not Serious	Very Low ⨁ⲞⲞⲞ	0.07 [−0.43, 0.56]	Small	No Favour

GRADE, Grading of Recommendations Assessment, Development and Evaluation; SMD, Standard Mean Difference; CI, Confidence Interval; CKCE, closed kinetic chain exercise.

^a^ Allocation concealment was not clearly reported; lack of blinding of participants or assessors and therapists; attrition bias due to incomplete outcome data,

^b^ Significant heterogeneity in meta-analysis, I^2^ > 75%,

^c^ Small sample size with wide confidence interval, participants less than 400

⨁⨁ⲞⲞ: low quality of evidence: The true effect might be markedly different from the estimated effect with limited confidence to the effect estimate.

⨁ⲞⲞⲞ: Very low quality of evidence: The true effect is probably markedly different from the estimated effect with very little confidence to the effect estimate.

### 3.7. Data synthesis

#### 3.7.1. Descriptive analysis.

Descriptive analysis was performed for all included studies, and the main findings are reported in Table 1.

#### 3.7.2. Meta-analysis.

Eighteen studies were included in the meta-analysis. Six studies were excluded due to missing information (e.g., mean and standard deviation for both the experimental and control groups) [18,25,30,32,40,41].

**Fig 2:**
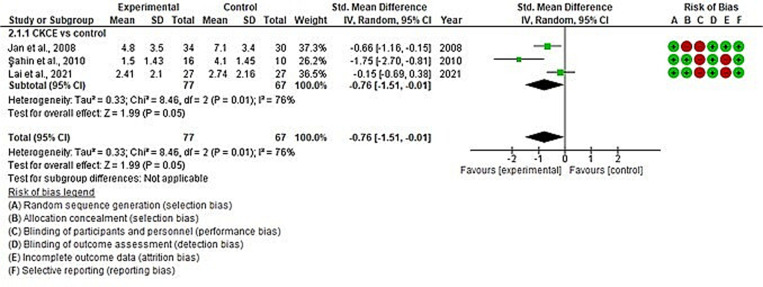
Forest plot of meta-analysis for the effects of CKCE vs. no treatment on pain intensity.

**Fig 3:**
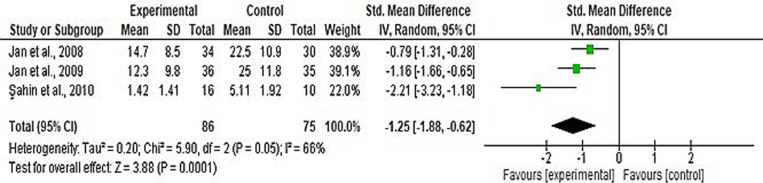
Forest plot of meta-analysis for the effects of CKCE vs. other treatments on pain intensity.

The overall effect size of CKCE (experimental) vs. no treatment (control) on pain reduction was analysed in three studies [21,27,28], and CKCE vs. other treatments was analysed in 12 studies [17,19,20,28,29,34,35,37–39,42,43]. The overall meta-analysis showed that CKCE significantly reduced pain compared to no treatment (SMD = −0.76; 95% CI: −1.51, −0.01; I² = 76%; *P* < 0.05; Fig 2), but there was no significant difference between CKCE and other treatments (SMD = −0.18; 95% CI: −0.55, 0.19; I² = 78%; *P* = 0.34; Fig 3).

**Fig 4:**
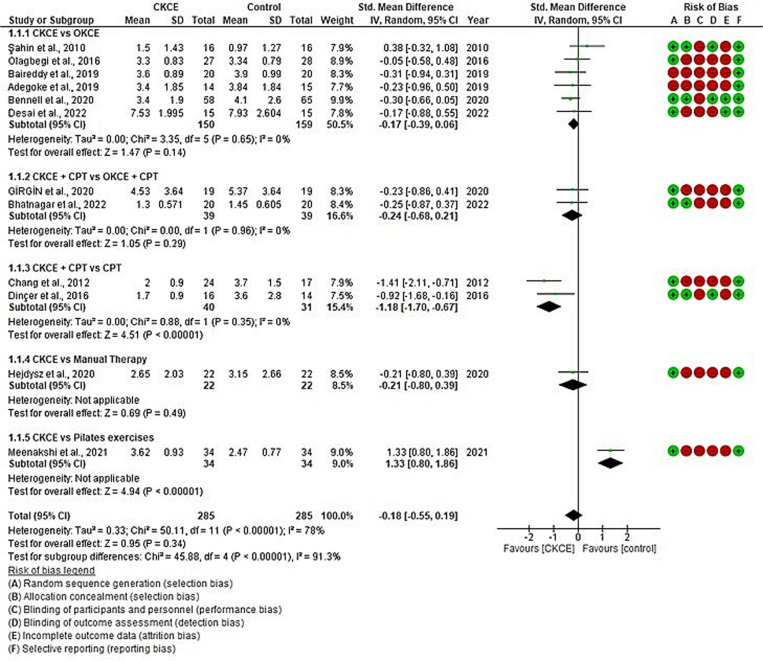
Forest plot of meta-analysis for the effects of CKCE vs. no treatment on functional level (WOMAC; the lower the score, the better the result/function).

**Fig 5:**
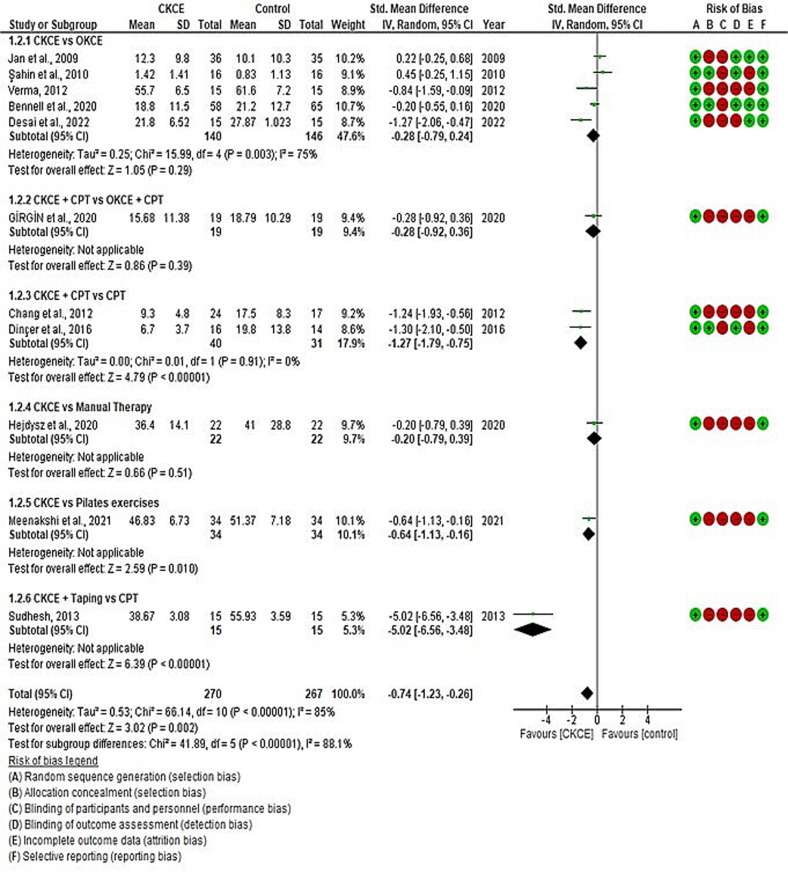
Forest plot of meta-analysis for the effects of CKCE vs. other treatments on functional level (WOMAC; the lower the score, the better the result/function).

**Fig 6:**
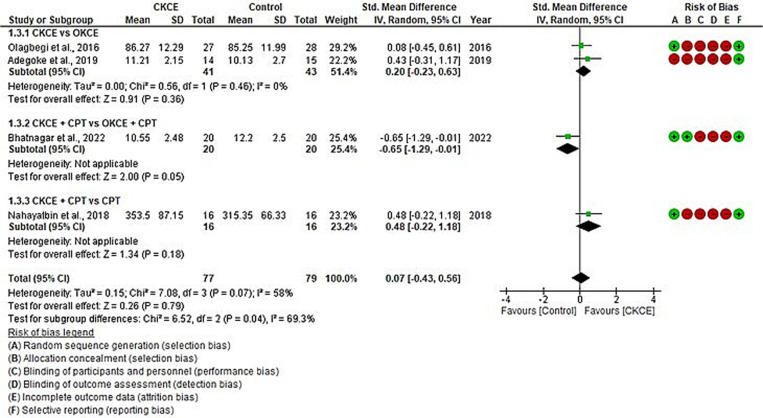
Forest plot of meta-analysis for the effects of CKCE vs. no treatment on functional level (IKHOAM, FIQ and KOOS; the higher the score, the better the result/function).

Functional improvement (WOMAC, the lower the score, the better the result/function) was assessed in three studies comparing CKCE vs. no treatment [26–28] and in 11 studies comparing CKCE vs. other treatments [20,26,28,29,31,33,34,37–39,43]. The overall meta-analysis revealed significant improvement in function for CKCE vs. no treatment (SMD = −1.25; 95% CI: −1.88, −0.62; I² = 66%; *P* < 0.0001; Fig 4) and CKCE vs. other treatments (SMD = −0.74; 95% CI: −1.23, 0.26; I² = 85%; *P* = 0.002; Fig 5). Four studies [17,35,36,42] employed different scales (the higher the score, the better the result/function) to evaluate the improvement of function such as IKHOAM, FIQ and KOOS. For these other scales of function, the overall meta-analysis revealed no significant difference between CKCE and other treatments (SMD = 0.07; 95% CI: −0.43, 0.56; I² = 58%; *P* = 0.79; Fig 6).

#### 3.7.3. Subgroup meta-analysis.

Subgroup meta-analyses showed significant results in certain comparisons. For example, CKCE + CPT significantly reduced pain [29,34] compared to CPT alone (SMD = −1.18; 95% CI: −1.70, −0.67; I² = 0%; *P* < 0.00001; Fig 3). Similar findings were observed for functional improvement [29,34], where CKCE + CPT provided better results than CPT alone (SMD = −1.27; 95% CI: −1.79, −0.75; I² = 0%; *P* < 0.00001; Fig 5). However, no significant differences were observed between CKCE and other treatments like OKCE or manual therapy in either pain or function outcomes.

## 4. Discussion

This systematic review and meta-analysis yielded several important findings. First, the meta-analysis indicates that CKCE has a medium effect size in reducing pain and a large effect size in improving function in individuals with knee OA compared to no treatment. Second, the meta-analysis also suggests that CKCE, either alone or in combination with other treatments, has a medium effect size in improving function compared to treatments without CKCE. Third, the subgroup meta-analysis indicates that CKCE combined with CPT has a large effect size in improving function compared to CPT alone. Fourth, the risk of bias assessment revealed that more than half of the included studies were of high quality according to the PEDro scale. However, the GRADE assessment indicates low-quality evidence regarding the effects of CKCE (alone or combined with other treatments) on pain reduction and functional improvement.

Evidence-based guidelines for treating knee OA endorse both land- and water-based CKCE [9–14]. CKCE is more effective because it encourages the use of lower limb muscles during daily activities, facilitating more effective contractions, including both eccentric and co-contractions, with simultaneous segmental movement [31]. CKCE predominantly involves eccentric muscular work, which increases muscle tension [44]. Additionally, CKCE minimizes shear forces while increasing compression forces and controlling the chain of motion across the joint, improving joint stability [31]. Therefore, CKCE is considered an effective functional exercise that enhances muscle strength and proprioception.

The results of this meta-analysis reveal significant improvements in pain and function with CKCE treatment compared to no treatment. This finding is based on the pooled results of three studies [26–28], which demonstrate CKCE as an effective treatment for pain reduction in individuals with knee OA. Reducing pain intensity and improving function may enhance the ability to perform ADLs, such as walking, increasing gait speed, and climbing stairs [27]. However, not all studies in the meta-analysis reported significant effects of CKCE compared to no treatment [21]. Discrepancies in results may be due to differences in treatment protocols between studies, where increased exercise intensity may have exacerbated pain.

In the subgroup meta-analysis, both CKCE and OKCE showed significant pain reduction, but the differences in magnitude between CKCE and OKCE were not significant [17,19,20,28,35,43]. A similar finding was observed when comparing CKCE + CPT to OKCE + CPT, where both treatment groups experienced significant pain reduction, but there was no significant difference between them [37,42]. However, CKCE + CPT had a significantly greater effect on pain reduction compared to CPT alone [29,34], suggesting that incorporating CKCE into conventional rehabilitation programs provides additional benefits. The subgroup meta-analysis also showed no differences in pain reduction between CKCE and other interventions, such as manual therapy. However, this finding may be limited by the small number of available studies [38] and the small sample sizes, which may have reduced the statistical power needed to detect differences between groups.

For functional improvement (WOMAC), subgroup meta-analysis indicated that both CKCE and OKCE had a similar beneficial effect on function, with no significant differences between the two treatments [20,26,28,31,43]. However, the evidence leaned in favour of CKCE, as two studies favoured CKCE over OKCE [31,43], and three studies [20,26,28] found comparable results between the two treatments in terms of functional improvement. The additional benefit of CKCE might be explained by its resemblance to many ADLs, such as sitting up, stair climbing, and squatting. Incorporating these exercises into the training program may better facilitate these functional activities than other exercises like OKCE. When CKCE + CPT was compared to OKCE + CPT, both treatments showed significant functional improvements, but no significant differences were found between them [37]. However, CKCE + CPT significantly improved function compared to CPT alone [29,34]. Similar to pain reduction, no significant functional differences were found between CKCE and manual therapy [38]. For functional scales other than WOMAC, the overall meta-analysis showed no differences between CKCE and other treatments [17,35,36,42].

Although meta-analysis for joint proprioception could not be performed due to missing required data (e.g., means and standard deviations), three studies investigated joint proprioception and compared CKCE to no treatment [21,25,26]. Two studies [25,26] found a significant difference between CKCE and the control, while one [21] reported no significant difference.

In general, there were serious inconsistencies when comparing CKCE with no treatment (for pain) and when comparing CKCE with other treatments for pain and function (e.g., WOMAC). These inconsistencies may be due to differences in sample size, intervention type and duration, number of sessions, exercise intensity, and progression.

Several limitations may affect the generalizability of the results, and the current evidence (GRADE) indicates low-quality evidence for the effects of CKCE on pain and function in individuals with knee OA. This could be due to several factors: (1) most studies had small sample sizes, (2) the type and intensity of training were inconsistent between studies, (3) the percentage of total variability due to between-study heterogeneity was moderate to high, and (4) most studies lacked long-term follow-up data. These factors may have impacted the accuracy of the results, making it difficult to establish clinical recommendations based on the existing evidence. Therefore, high-quality trials are needed.

## 5. Conclusion

Although CKCE shows promise as a treatment for individuals with knee OA in terms of pain reduction and functional improvement, the low quality of the studies limits confidence in the effect estimates. Further long-term, high-quality RCTs with large sample sizes are required.

## Supporting information

S1 Table PRISMA checklist. (DOCX)

S2 Table Inclusion criteria. (DOCX)

S3 Table Search strategy in each database. (DOCX)

S4 Table Study selection and inclusion/exclusion criteria through primary sources. (XLSX)
