# Supplementary material for: The Effectiveness of closed kinetic chain exercises in individuals with knee osteoarthritis: A systematic review and meta-analysis
Source: PLoS One. 2025 May 2;20(5):e0322475. doi: 10.1371/journal.pone.0322475 (PMC12047827; doi:10.1371/journal.pone.0322475)
Supplement: S2 Table — (DOCX) [file pone.0322475.s002.docx]

**Table S1. Inclusion criteria**

| **PICOS** | **Description** |
| --- | --- |
| Population (P) | Participants who are suffering from knee osteoarthritis regardless of gender, race, educational level, and economic condition |
| Interventions (I) | Closed kinetic chain exercises alone or combined with other therapy |
| Comparisons (c) | Any other type of exercise or physical activity (e.g., open kinetic chain exercise or aerobic exercise) and another treatment method (e.g., pharmaceuticals, taping, electrical stimulation) |
| Outcomes (O) | Primary outcomes: Pain  Secondary outcomes: Function |
| Study design (s) | Randomized controlled trials or clinical trials |
