# Supplementary material for: The Effectiveness of closed kinetic chain exercises in individuals with knee osteoarthritis: A systematic review and meta-analysis
Source: PLoS One. 2025 May 2;20(5):e0322475. doi: 10.1371/journal.pone.0322475 (PMC12047827; doi:10.1371/journal.pone.0322475)
Supplement: S3 Table — (DOCX) [file pone.0322475.s003.docx]

**Table S2. Search strategy in each database**

| **Database** | **No. of papers** | **Search Strategy** |
| --- | --- | --- |
| PubMed | 121 | **Search:** (((Resistance training [MeSH Terms]) OR (Strength Training [Text Word]) OR (Strengthening exercise [Text Word]) OR (closed kinetic chain exercise [Text Word]) OR (weight-bearing exercise [Text Word])) AND ((knee osteoarthritis [MeSH Terms]) OR (knee osteoarthrosis [Text Word]) OR (knee OA [Text Word]) OR (osteoarthritis of knee [Text Word]) OR (knee joint degenerative disease [Text Word])))  **Filters:** Article Type (Clinical Trial, Randomized Controlled Trial), Species (Humans), Language (English), year (2000-2022). |
| Medline | 136 | **Search:** ((Resistance training or Strength Training or Strengthening exercise or closed kinetic chain exercise or weight-bearing exercise) AND (knee osteoarthritis or knee osteoarthrosis or knee OA or osteoarthritis of knee or knee joint degenerative disease))  **Limits:** year (2000-2022), Article Type (Clinical Trial, Randomized Controlled Trial), Language (English), Species (Humans). |
| Cochrane Central Register of Controlled Trials (CENTRAL) | 131 | **Search:** (Osteoarthritis, Knee [MeSH]) AND (Resistance Training [MeSH] OR closed kinetic chain exercise [ti,ab,kw] OR weight-bearing exercise [ti,ab,kw])  **Limiters:** Publication Year (2000-2022), Content type (Trials)  **Expanders:** Word variations have been searched |
| Web of science | 158 | **Search:** [closed kinetic chain exercise (Title) OR weight-bearing exercise (Title) OR Resistance Training (Title) OR closed kinetic chain exercise (Title) OR closed kinetic chain exercise (Abstract) OR weight-bearing exercise (Abstract) OR Resistance Training (Abstract)] AND [ knee osteoarthritis (Title) OR knee OA (Title) OR knee joint degenerative disease (Title) OR knee osteoarthritis (Abstract) OR knee OA (Abstract) OR knee joint degenerative disease (Abstract)]  **Filters:** Document Types (Article), Languages (English), Publication Date (2000-2022) |
| EBSCO | 395 | **Search:** (Resistance training OR Strength Training OR Strengthening exercise OR closed kinetic chain exercise OR weight-bearing exercise) AND (knee osteoarthritis OR knee osteoarthrosis OR knee OA OR osteoarthritis of knee OR knee joint degenerative disease) AND (randomized controlled trial or clinical trial)  **Limiters:** Published Date (2000-2022), Language (English), Source Types (Academic Journals)  **Expanders:** Apply related words |
| Wiley Online Library | 148 | **Search:** "“knee osteoarthritis” OR “knee OA” OR “knee joint degenerative disease”" and "“Resistance training” OR “closed kinetic chain exercise” OR “weight-bearing exercise”"  **Filters:** Publication Date (2000 – 2022), Publication Type (Journals) |
| Science Direct | 646 | **Search:** (Closed kinetic chain exercise OR weight-bearing exercise) AND (knee osteoarthritis OR knee joint degenerative disease) AND (randomized controlled trial or clinical trial)  **Filters:** Year (2000-2022), Article type (Research articles) |
| CINAHL | 178 | **Search:** (Resistance training OR Strength Training OR Strengthening exercise OR closed kinetic chain exercise OR weight-bearing exercise) AND (knee osteoarthritis OR knee osteoarthrosis OR knee OA OR osteoarthritis of knee OR knee joint degenerative disease) AND (randomized controlled trial or clinical trial)  **Limiters:** Published Date (2000-2022), Language (English), Source Types (Academic Journals)  **Expanders:** Apply related words |
| PEDro | 10 | **Search:** Abstract &title (Knee osteoarthritis AND closed kinetic chain exercise)  **Filters:** Method (clinical trial), Published since (2000) |
|  | 13 | **Search:** Abstract &title (Knee osteoarthritis AND weight-bearing exercise)  **Filters:** Method (clinical trial), Published since (2000) |
